# Metrological Comparison of Available Methods to Correct Edge-Effect Local Plasticity in Instrumented Indentation Test

**DOI:** 10.3390/ma16124262

**Published:** 2023-06-08

**Authors:** Jasurkhuja Kholkhujaev, Giacomo Maculotti, Gianfranco Genta, Maurizio Galetto

**Affiliations:** 1 Department of Management and Production Engineering, Politecnico di Torino, Corso Duca degli Abruzzi 24, 10129 Turin, Italy; jasurkhuja.kholkhujaev@polito.it (J.K.); gianfranco.genta@polito.it (G.G.); maurizio.galetto@polito.it (M.G.); 2 Department of Mechanical and Aerospace Engineering, Turin Polytechnic University in Tashkent, Kichik Halka Yuli, 17, Tashkent 100095, Uzbekistan

**Keywords:** nanoindentation, pile-up, measurement uncertainty

## Abstract

The Instrumented Indentation Test (IIT) mechanically characterizes materials from the nano to the macro scale, enabling the evaluation of microstructure and ultra-thin coatings. IIT is a non-conventional technique applied in strategic sectors, e.g., automotive, aerospace and physics, to foster the development of innovative materials and manufacturing processes. However, material plasticity at the indentation edge biases the characterization results. Correcting such effects is extremely challenging, and several methods have been proposed in the literature. However, comparisons of these available methods are rare, often limited in scope, and neglect metrological performance of the different methods. After reviewing the main available methods, this work innovatively proposes a performance comparison within a metrological framework currently missing in the literature. The proposed framework for performance comparison is applied to some available methods, i.e., work-based, topographical measurement of the indentation to evaluate the area and the volume of the pile-up, Nix–Gao model and the electrical contact resistance (ECR) approach. The accuracy and measurement uncertainty of the correction methods is compared considering calibrated reference materials to establish traceability of the comparison. Results, also discussed in light of the practical convenience of the methods, show that the most accurate method is the Nix–Gao approach (accuracy of 0.28 GPa, expanded uncertainty of 0.57 GPa), while the most precise is the ECR (accuracy of 0.33 GPa, expanded uncertainty of 0.37 GPa), which also allows for in-line and real-time corrections.

## 1. Introduction

**Figure 1:**
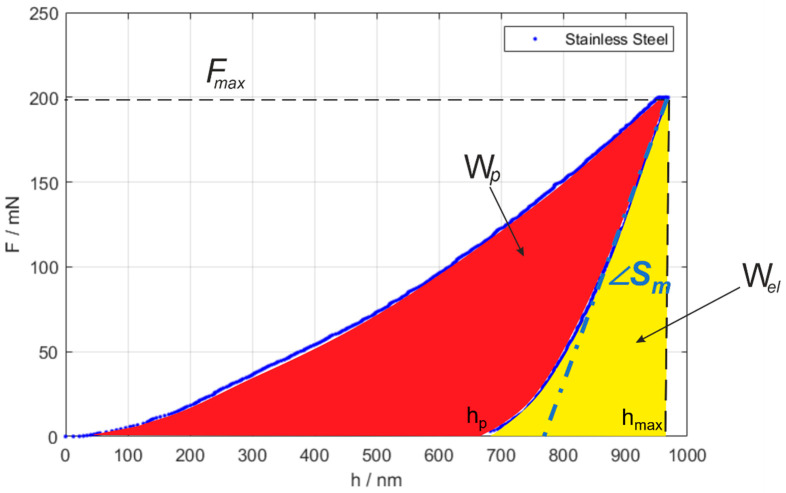
Example of indentation curve (IC), highlighting the main parameters: first contact point *h_0_*, maximum penetration *h_max_* and force *F_max_*, residual plastic penetration *h_p_*, total measured contact stiffness *S_m_* and the plastic (red area) and elastic (yellow area) work, *W_p_* and *W_el_*, respectively.

The Instrumented Indentation Test (IIT) is a non-conventional mechanical characterization method based on a depth-sensing hardness measurement technique [1]. The method, standardized in ISO 14577 [2], applies a loading–holding–unloading force-controlled cycle on a test sample by an indenter of known geometry. Measuring the applied force *F* and the resulting penetration depth *h* allows resolving mechanical characteristics at the nanoscale. IIT was conceived as a hardness-testing technique, such that the indentation hardness *H_IT_* is defined as [2]:

(1) $$H_{IT}=\frac{F_{max}}{A_{p}(h_{c,max})}$$

i.e., the ratio between the maximum applied force and the area of the contact surface between the indenter and the test sample projected on the plane is normal to the force application direction. Furthermore, the analysis of the indentation curve (IC), i.e., *F(h)* as shown in Figure 1, allows the evaluation of other mechanical properties, such as the indentation modulus *E_IT_* estimating the Young’s modulus, and the indentation creep. Additionally, further mechanical properties can be evaluated by convenient modification of the quasi-static indentation cycle. The replication of quasi-static indentations in the same location at increasing load enables the evaluation of the material properties as a function of the increasing penetration depth [3], allowing coatings [4], organic coatings [5] and surface treatment characterization [6], without cross-sectioning. Dynamic indentations, obtained by superimposing a sinusoidal frequency to the applied load, allow evaluating the damping properties of the material, which are particularly relevant for polymers [7,8]. Displacement-controlled indentation cycles allow estimating relaxation properties [2,8].

Moreover, IIT can be effectively deployed to map surface properties, quantitatively distinguishing among different phases of materials (both polycrystalline [9] and composites [10,11]), and estimate critical grain size dimensions [12].

Last, the identification of discontinuities in the indentation curve is expedient to highlight phase changes and penetration of coating or oxide layers [13]. Data augmentation via electrical contact resistance (ECR) further enhances IIT, enabling the critical loads to induce phase change for semiconductors, e.g., silica, and germanium [14,15].

Therefore, IIT finds applications in several strategic industrial and research fields, e.g., automotive, aerospace, physics and military, and it supports the development of innovative materials, such as shape-memory alloys [16], metallic glasses [17] and glass-fiber-reinforced polymers [18], and manufacturing processes. For example, the mechanical properties of coatings for batteries for e-mobility are related to efficiency and durability [19]. Freeform manufacturing of germanium and semiconductors is essential for night vision, which finds application in physics and military, and for solar-panel coating efficiency [20,21].

Accordingly, ensuring high accuracy of the IIT characterization results is of utmost importance. Systematic error correction is achieved by ensuring a controlled temperature environment and the stabilization of the contact conditions [1,22], and by removing elastic contribution to the indenter displacement due to the machine frame compliance *C_f_* [23,24,25], and the sample contact stiffness *S* [25,26,27], and possible zero errors due to the first contact *h_0_* [28]:

(2) $$h_{c,max}=h_{max}-h_{0}-C_{f}F_{max}-\varepsilon \frac{F_{max}}{S}$$

(3) $$\frac{1}{S}=\frac{1}{S_{m}}-C_{f}$$

(4) $$S_{m}={\left.\frac{\partial F}{\partial h}\right|}_{h_{max}}$$

where ε is a parameter dependent on the indenter geometry. The frame compliance requires calibration [23,24,25] and ensures traceability.

Furthermore, geometrical errors in the indenter geometry are addressed by calibrating the area shape function, i.e., $A_{p}(h_{c,max})$, either by direct [29,30,31] or indirect methods [23,24,32].

**Figure 2:**
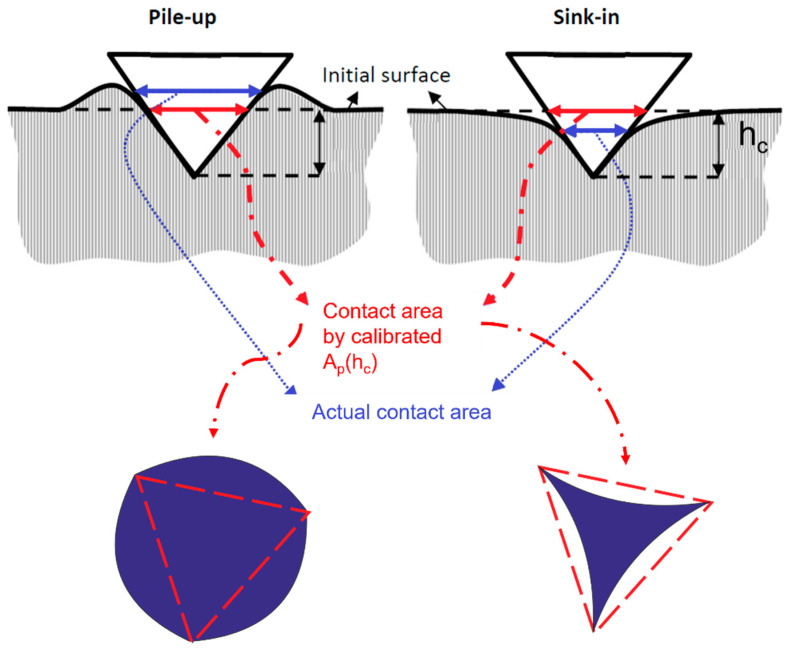
Edge effect: pile-up and sink-in. Note how the calibrated area shape function respectively underestimates and overestimates the actual contact area.

Additional sources of bias are due to the physics of the indentation. Specifically, two errors may be generated. The first is the so-called indentation size effect (ISE). The ISE is due to geometrically necessary dislocations which generate an apparent increase in the material hardness as soon as the indentation size approximates the grain dimensions [33,34]. ISE can be exploited to determine grain dimension, and hardness correction can then follow [33,35]. The second is due to the edge effect, i.e., localized plasticity at the indentation edges, see Figure 2.

Edge effects induce the material to either pile up or sink in at the indentation edge. This results in a biased estimation of the contact area by the calibrated area shape function. Specifically, it is underestimated in the case of pile-up and overestimated when sink-in occurs. Edge effects are induced by material plasticity and mechanical response. As shown in the seminal work of Cheng and Cheng [35], materials showing large yield strength to the Young modulus ratio are characterized by sink-in, i.e., materials with a mainly elastic behavior. Conversely, for a larger ratio, the material response is affected by the work hardening (*n*). Severely work-hardened materials (*n*~0.5) still tend to sink in, whilst materials approaching an almost ideally plastic behavior (*n* < 0.1), e.g., copper and mild steels, show pile-up. Therefore, prior stress and strain aging affect the phenomenon [36]. Edge effect is quite common in several materials [37], biasing results of both conventional steel grades, e.g., for spur gear manufacturing [38], and for deep drawing applications [39], and advanced materials, both polycrystalline [34,40,41] and monocrystalline [42].

### 1.1. Edge-Effect Correction Methods

The management of the edge effect is traditionally extremely challenging for hardness measurements. Historically, Vickers hardness was introduced, which is based on diagonal measurements, more robust to edge plasticity, to relieve the effects of edge effect in the case of spherical indenters [43]. Furthermore, at larger characterization scales typical of Vickers and Brinell hardness tests, the error introduced by the edge effect is negligible [24,44,45]. However, the need to resolve nanoscale hardness and the possibility of characterizing additional mechanical properties by IIT, made it necessary to find approaches to correct the systematic error introduced by the presence of non-negligible edge effects.

The literature presents several approaches to predict and correct edge effects. These can be classified, as in Table 1, as based on work, topographical methods and parameters evaluated from the analysis of the IC, FEM, ISE and ECR modeling. Indeed, they feature different generalization capabilities and robustness. Furthermore, the degree to which a practical application can be performed varies. For example, while being traceable, topography-based approaches rely on external measurement systems that can be calibrated and require post hoc analysis of the indentation. This is particularly critical because it requires accurately locating the indentation, which is typically an order of magnitude smaller than the surface on which is performed, and feature resolution limitation or the liability of tip convolution, if scanning probe microscopes (SPM) are exploited. On the other hand, other approaches do not allow a traceable correction of the edge effect, as they exploit the same measurement system and no external reference but can be applied in real-time. Indeed, different models at increasing complexity may be more or less easily manageable, requiring specific expertise for a robust implementation. FEM-based methods hold a particular position. These, strictly speaking, do not allow a correction of the indentation, as they address inverse indentation problems, but rather allow insights on the elasto-plastic behavior of materials at a multi-scale level, which is essential to validate other approaches.

A more detailed summary and description of a representative correction solution as per literature, per each approach, is presented in the following. The specific solution is chosen considering the criteria of generality, robustness and ease of implementation.

#### 1.1.1. Oliver and Pharr (2004) (F/S^2^)

This method integrates work-based modeling and the analysis of the indentation curve [69]. Specifically, let the plastic work be *W_p_*, elastic work due to the elastic recovery *W_el_* and the total work *W_tot_*:

(5) $$W_{p}=\int_{h_{0}}^{h(t=end\;of\;hold)}F(h)dh$$

(6) $$W_{el}=\int_{h(t=end\;of\;hold)}^{h_{p}}F(h)dh$$

(7) $$W_{tot}=W_{p}+W_{el}$$

Cheng and Cheng [35] showed that the approximate relationship could be written between work and mechanical properties:

(8) $$\frac{W_{tot}-W_{el}}{W_{tot}}\sim 1-5\frac{H_{IT}}{E_{r}}$$

(9) $$\frac{1}{E_{r}}=\frac{1-\nu_{s}^{2}}{E_{s}}+\frac{1-\nu_{i}^{2}}{E_{i}}=\frac{2\sqrt{A_{p}(h_{c,max})}}{S\sqrt{\pi }}$$

where *E_r_* is the reduced modulus, the subscripts *s* and *i* indicate the sample under test and the indenter material, and ν is the Poisson ratio. It is worth noting that *E_r_* can be either evaluated from the IIT or obtained by calibrated values.

Furthermore, it is easy to demonstrate, considering Equations (1) and (9), that the following equation can be written and is constant (provided no biased measurements are performed) and independent from the area measurement:

(10) $$\frac{4F_{max}}{\pi S^{2}}=\frac{H_{IT}}{E_{r}^{2}}$$

Thus, combining Equations (8) and (9), an expression of the indentation hardness results that is independent of the area measurement is:

(11) $$H_{IT,c}=\frac{\pi }{100}\frac{S^{2}}{F_{max}}{\left(1-\frac{W_{p}}{W_{tot}}\right)}^{2}$$

The method presents a convenient integration of well-known and largely exploited work-based approaches to a more straightforward and easily accessible approach based on the IC analysis. Indeed, a possible limitation is that Equation (8) is an approximated relationship dependent on the work hardening coefficient and yield strength of the materials, shall it be computed exactly.

#### 1.1.2. Qiu (2018) (Area)

Surface topography measurement-based approaches can be summarized in estimating a corrected indentation hardness as:

(12) $$H_{IT,c}=\frac{F_{max}}{A_{p,c}}=\frac{F_{max}}{A_{p}+A_{edge\;effect}}$$

where the correction is provided by a topographical measurement of the corrected area, which can also be seen as a corrective term due to the edge effect for the projected contact area estimated from calibration.

Topographical measurements obtain a representation of the surface 𝒮(*x,y,z*) heights *z* as a function of (*x,y*) locations, i.e., 𝒮 = *z*(*x*,*y*). Measurements are performed considering a certain lateral sampling resolution *p_xy_*, typically dominated by the pixel size in the case of optical instruments or by linear encoders’ stepping resolution for SPM.

Several approaches are available in the literature. The most general are those based on numerical solutions, such that, once the edge-effect-affected topography has been segmented, the corrected area is:

(13) $$A_{edge\;effect}=\sum_{S_{edge\;effect}}p_{xy}$$

and is positive in case of pile-up or negative when sink-in occurs.

However, these methods may be complex, as they require non-trivial segmentation, mostly based on machine vision [80] to identify the geometries.

**Figure 3:**
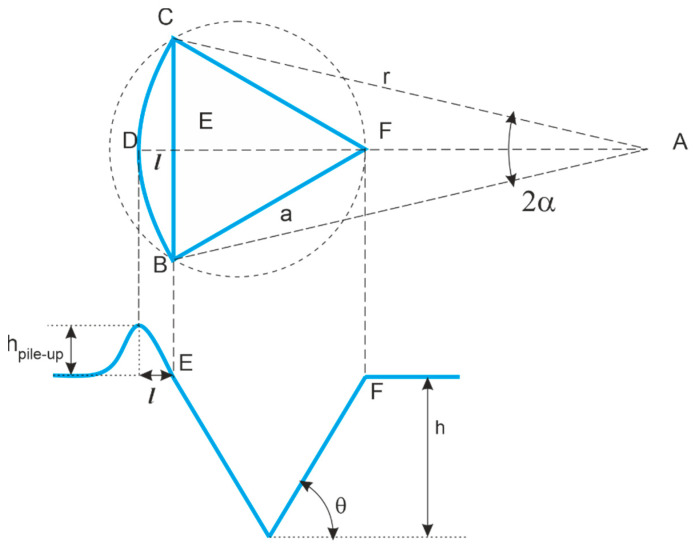
Quantity definition for the pile-up correction based on topographical measurement and geometrical description of the edge effect. Upper case letters correspond to geometry points, while lower case letters are used for empirical evaluations. *a* is a side length of the indented pyramid; *r* the radius of circle circumcising the projected pile-up arc BDC with center at A; *h* is indentation depth after elastic recovery; *h_pile-up_* the height of the pile up; θ the half-dihedral angle of the indenter.

Alternatives are based on a geometrical description of the edge-effect-affected zone. These have been proposed by [62] and later modified and refined [66]. With reference to Figure 3, we assume a Berkovich indentation BCF, with measured indentation height *h* and pile-up height *h_pile-up_*. The corrective area term can be evaluated per each side as:

(14) $$A_{edge\;effect}=\sum_{j=1}^{number\;of\;sides}\alpha_{j}r_{j}^{2}-\frac{a_{j}}{2}\left(r_{j}-l_{j}\right)$$

(15) $$a_{j}=7.53h$$

(16) $$\alpha_{j}=arsin\left(\frac{a_{j}}{2r_{j}}\right)$$

(17) $$l_{j}=\frac{h_{pile-up,j}}{tan\left(\theta \right)}$$

(18) $$r_{j}^{2}={\left(\frac{a_{j}}{2}\right)}^{2}+{\left(r_{j}-l_{j}\right)}^{2}\to r_{j}=\frac{{\left(\frac{a_{j}}{2}\right)}^{2}+l_{j}^{2}}{2l_{j}}$$

This approach is more robust to the identification of the edge-effect topography, as it only requires the evaluation of the indentation side *a* and the maximum pile up height *h_pile-up_*.

#### 1.1.3. Beegan et al. (2005) (W/V)

More recently, Beegan et al. [46] proposed a hybrid approach integrating the traditional work-based description of the pile-up with an additional external traceable reference obtained by surface topography measurements. In particular, the method considers the indentation hardness definition; see Equation (1). By simple modifications, it obtains a relationship depending on the ratio of the plastic work and the plastically displaced volume *V_p_*:

(19) $$H_{IT,c}=\frac{F_{max}}{A_{p}(h_{c,max})}=\frac{\int_{h_{0}}^{h(t=end\;of\;hold)}F(h)dh}{\int_{h_{0}}^{h(t=end\;of\;hold)}A_{p}(h)dh}=\frac{W_{p}}{V_{p}}$$

The plastically displaced volume can be obtained as the material volume *V_m_*, i.e., a surface topography volume parameter [81], computed considering the reference undeformed surface height z^*^ as the thresholding height:

(20) $$V_{p}=p_{xy}^{2}\int_{0\%}^{m_{r}^{*}}\left(S_{mc}\left(w\right)-S_{mc}\left(m_{r}^{*}\right)\right)dw$$

(21) $$S_{mc}\left(m_{r}^{*}\right)=z^{*}↔S_{mr}\left(z^{*}\right)=P\left[0<z<z^{*}\right]=m_{r}^{*}$$

where *m_r_* is the material ratio, i.e., the cumulated probability of the surface topography height distribution up a certain threshold value. The cumulated probability function is $S_{mr}$, i.e., the areal material ratio function (also known as the Abbot–Firestone curve), and $S_{mc}$ is its functional inverse [82].

#### 1.1.4. Indentation Size Effect (ISE)

As mentioned in the Introduction, ISE introduces an apparent material hardening at small penetration depths, as geometrically necessary dislocations are added to the statistically stored dislocation to cope with the geometric singularity and high local deformation introduced by the indentation [33]. The phenomenon has been modeled as:

(22) $$H_{IT,c}^{2}=H_{0}^{2}\left(1+\frac{h^{*}}{h_{c,max}}\right)$$

which relates the measured indentation hardness $H_{IT}$ to the bulk hardness $H_{0},$ thus unaffected by ISE. The model can predict corrected hardness at large scales, while considering a scaling effect. In fact, the prediction model presents a parameter $h^{*}$ which is a critical length, dependent on the material properties of the indentation pair (sample and indenter) and the indenter geometry. In a $\frac{1}{h_{c,max}};H_{IT}^{2}$ plane, the model is linear, and the intercept is $H_{0}^{2}$. The representation is particularly useful because deviations from linearity at small $\frac{1}{h_{c,max}}$ indicate an edge effect, which is not included in the model.

#### 1.1.5. Electrical Contact Resistance (ECR)

**Figure 4:**
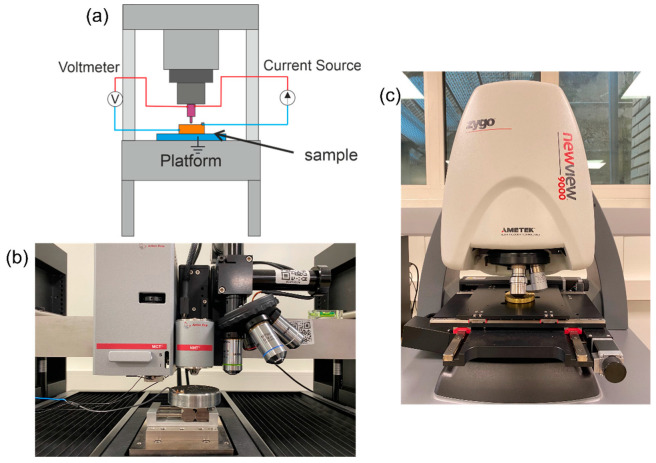
(**a**) Schematic of ECR set-up. (**b**) Anton Paar MCT3 and NHT3 with wiring for ECR. (**c**) CSI Zygo NewView9000.

The approach based on ECR is the most recently introduced [79]. ECR was originally conceived to detect phase transformation in semiconductors and different phases in composites [83]. It consists of augmenting IIT by in situ electrical measurements obtained using a conductive doped-diamond indenter. Typically, a current-controlled circuit is created, and the resulting voltage between the indenter and the sample is measured (see Figure 4a). The fundamental relationship that is exploited is the well-known dependence of the resistance on the area of the conductive medium. Considering the contribution due to the system electronics *R_0_*, the contact resistance (predominant at contact onset), and the spreading resistance (approximating the bulk material resistance and depending on the contact pair resistivity), a corrected projected contact area can be obtained:

(23) $$R=R_{c}+R_{s}+R_{tip}+R_{electronics}=\frac{C_{1}}{A_{p}}+\frac{C_{2}}{\sqrt{A_{p}}}+R_{0}$$

(24) $$A_{p}(R)=\frac{C_{3}}{\sqrt{R}}+\frac{C_{4}}{R^{2}}+C_{5}$$

The parameters of Equation (24) can be obtained by calibration on a material that, at the characterization scale, does not present a significant edge effect, e.g., aluminum alloys or brass [79]. Indeed, the calibrated parameters are material-dependent. Thus, to enable the edge-effect correction on any other material, indentation should be performed on a wide range of forces, including both measurement scales affected and unaffected by edge effect. The data collected at the scales not affected by edge effect can be exploited to normalize Equation (24) considering a first-order approximation, i.e., $A_{p}\left(∆R=R-R_{0}\right)\approx \frac{C_{4}}{{∆R}^{2}}$, holding at the edge-effect scales. Normalized data for the material needing correction (indicated with subscript $m_{C}$) are obtained:

(25) $$A_{p,m_{C},corr}\left({\Delta R}_{m_{C}}^{*}\pm \delta R\right)=A_{p,m_{R}}(\Delta R_{m_{C}}^{*}\pm \delta R)\frac{A_{p,m_{C}}(h_{c,m_{C}}^{*})}{A_{p,m_{C}}({\Delta R}_{m_{C}}^{*})}$$

where the subscript $m_{R}$ indicates the reference material, $A_{p,m_{R}}$ is the calibrated relationship in Equation (24), and the asterisk (*) indicates quantities collected at scales unaffected by edge effect. Because the approximation holds in small intervals δR, from the normalized data, a new regression for the material needing correction can be re-evaluated to predict edge-effect correction [79].

The ECR approach has the advantage of allowing in-line correction, but it is limited to conductive materials.

### 1.2. Scope of the Work

Within such a complex framework, identifying adequate correction models is extremely difficult. The literature reports some attempts of comparison, but they are limited in their scope and neglect metrological performances of the methods in terms of accuracy and precision. In fact, it is customary to perform comparisons when novel approaches are introduced. However, in the best-case scenario, such comparisons only focus on similar alternatives, thus being limited in their scope and conclusions. Typically, methods are validated with FEM and benchmarked against topographical methods. Furthermore, a metrological framework is currently missing. Therefore, this work aims at providing a comparison of the different methods, addressing the measurement uncertainty of the correction while evaluating the accuracy and the precision of the edge-effect correction methods. First, the methods were classified. Then, amongst the available methods, the more practical were considered for the comparison, whose metrological foundation is innovatively presented in this work. Section 2 presents the experimental set-up and the methodology to evaluate the measurement uncertainty and accuracy, which is currently missing in the literature. Section 3 presents results that are discussed in Section 4. Finally, Section 5 draws conclusions.

## 2. Materials and Methods

### 2.1. Experimental Set-up

This work compares the edge-effect correction method presented in Section 1.1 within a metrological framework. Indentations were performed on a stainless-steel sample hardness reference block, calibrated by the macro IIT indentation platform ASHU09 by AXIOTEK (Induno Olona, Italy), with a calibrated indentation hardness of (7.30 ± 0.355) GPa, stated with uncertainty at a 95% confidence level (evaluated on *n_cal_* = 40 replicated indentations). The calibration by macro-IIT was performed by considering ten replicated indentations at four different maximum loads, i.e., (300, 400, 500, 600) N. The force-controlled cycles were performed with a constant force gradient and duration of the loading, holding and unloading phase all of 30 s. The hardness block is a typical reference hardness block, calibrated for (64.3 ± 0.1) HRC, manufactured and commercialized by Mitutoyo (Torino, Italy) and HRC-calibrated by INRiM. The experimental plan is limited to demonstrating the performance comparison methodology to only one material, considering an industrially relevant application, i.e., stainless steel, which is also typically used to manufacture hardness blocks for indirect verification and calibration of test platforms for Rockwell, Brinell and Vickers hardness scales.

Instrumented indentation tests to compare performances of the edge-effect correction methods were performed with a state-of-the-art indentation platform, STeP6 by Anton Paar (Neuchatel, CH), featuring an MCT^3^ indenter head (calibrated force transducer with 0.5 mN of measurement uncertainty and LVDT displacement sensor having a relative expanded uncertainty of 0.6%) and an NHT^3^ indentation head (piezoelectric force-displacement transducer with relative expanded uncertainty of 1%). The indentation set-up is shown in Figure 4b. The indentation platform is hosted in the metrological room of the Mind4Lab @ the DIGEP-Politecnico di Torino and mounts a modified Berkovich indenter (Neuchatel, CH). The frame compliance and the area shape function were calibrated per ISO 14577-2 method 4 in Annex D with certified reference material by NPL (SiO_2_ and W) [31]. Ten replicated indentations were performed at (0.2, 0.3, 0.5, 0.7, 1, 1.5, 2, 3, 4, 5, 6, 7, 8, 9, 10, 12, 15, 17, 20, 23, 25, 30) N. All indentation cycles were force-controlled, with constant force gradient, and duration of the loading, holding and unloading phase of 30 s each. The investigated range is such to elicit ISE at low forces and a significant pile-up at high forces.

Data augmentation to apply the ECR method (Section 1.1.5) was obtained by in-house prototyping of the system, performed under the supervision of the instrument manufacturer (TriTec-Anton Paar), with a SIGLENT SPD3303X (Torino, Italy) current generator (accuracy of 0.5%) set at 10 mA and a maximum voltage of 6 V. The ECR features a current-controlled circuit to avoid overcharging the system and generating electrical arcs between the sharp indenter tip and the conductive metal surface of the sample. The creation of electrical arcs should be avoided, as it might wear and damage the indenter tip, and might induce, due to high localized heating, change in the microstructure of the tested sample, thus biasing the characterization results.

Data to apply surface topography measurement-based correction of the pile-up (Section 1.1.2 and Section 1.1.3) were collected by measuring each indentation with a state-of-the-art Coherence Scanning Interferometer (CSI) Zygo NewView9000 (Milano, Italy) hosted at the metrological room of the Mind4Lab@PoliTO, see Figure 4c. The CSI was equipped with a 50× Mirau objective, with a numerical aperture of 0.52, a field of view of (0.17 × 0.17) mm and a squared pixel of (0.17 × 0.17) µm. The CSI metrological characteristics [84] were calibrated, resulting in noise and flatness standard uncertainty of 1 nm and linearity and amplification on the *z*-axis of 10 nm. Uncertainty on the horizontal axis is dominated by lateral resolution due to the pixel size of 0.1 µm. The measured surface topographies were removed from noise by the application of a standard robust Gaussian S-filter, with nesting index of 0.5 µm. Subsequently the plane deviation was corrected by least-square plane fitting through the F-operator. L-filter application to remove waviness was not applied to avoid removing relevant topographical scales to the plastically displaced material.

### 2.2. Metrological Performance Evaluations

This work aims at a performance comparison within a metrological framework. Thus, the evaluation of the accuracy and precision of each edge-effect correction method will be assessed. Let us consider the *n* force levels needing correction (indicated with the subscript *j*), each containing *q* replicated measurements. Accordingly, after the correction, per each of those groups, the average HIT,c,j and the variance $s_{H_{IT,c,j}}^{2}$ can be evaluated.

The accuracy of each edge effect correction method is evaluated as the RMSE with respect to the calibrated reference value, i.e.,:

(26) Acc=∑j=1nHIT,c,j−HIT,calibrated2n

The precision is evaluated as the measurement uncertainty. The uncertainty of the different models is estimated according to the Guide to expression of Uncertainty in Measurement (GUM) [85], and applying the law of uncertainty propagation:

(27) uHIT,c,j=∑k=1Kck2uxk,j2+sHIT,j2=∑k=1K∂HIT,c(x)∂xkxk=xk,j2uxk,j2+sHIT,j2

(28) $$U\left(H_{IT,c,j}\right)=t_{0.975,dof}^{}\cdot u\left(H_{IT,c,j}\right)$$

here explicated for the corrected hardness $H_{IT,c,j}$ at the *j*th force level, where $x_{k}$ are the influence factors to each corrected indentation hardness model, $c_{k}$ is the sensitivity coefficient, and $s_{H_{IT,j}}^{2}$ is the reproducibility of the replicated measurement. Indeed, the evaluation is performed independently for each evaluation load. Equation (28) explicates the expanded uncertainty, evaluated with a coverage factor as the quantile of the Student’s *t* distribution associated with a cumulated probability of 0.975, and a certain number of degrees of freedom (*dof*) that can be evaluated by the Welch–Satterthwaite formula [85].

Standard uncertainty contribution of IIT-measured quantities, i.e., *F*, *h*, *S*, considers reproducibility (evaluated from the replicated tests), resolution and calibrated accuracy as relevant metrological characteristics.

Work (*W_p_* and *W_el_*) and volume (*V_p_*) uncertainty are obtained by applying Equation (17) to their definition (Equations (3) and (20)), which can be solved numerically by rectangle approximation [82]. The metrological characteristics of the measurement scales, i.e., *F*, *h*, for the work and the motion axes for the volume, are propagated.

Geometrical quantities necessary for applying topographical methods (Section 1.1.2) are considered affected by the relevant metrological characteristics of the surface topography measuring instrument and the measurement reproducibility evaluated from the replicated indentation measurements.

The management of ISE- and ECR-based method is more straightforward, as they are based on regression methods. As far as the ISE model correction approach is concerned, the model intercept estimates $H_{IT,c}^{2}$. Thus, knowing the standard error of the intercept (${SE}_{H_{IT,c}^{2}}$), the uncertainty can be evaluated accordingly by applying Equation (27). Conversely, for the ECR-based approach, the measurement uncertainty is simply the prediction interval of the scaled and normalized regression [79,86].

The evaluation of the accuracy and the measurement uncertainty allows assessing statistically significant differences in correction methods, which is currently disregarded by the literature.

Specifically, hypothesis tests based on Student’s *t* can be performed to compare the corrected values and the reference value [86]. Assuming as a null hypothesis that the average of the corrected data (for the *j*th force) is equal to the calibrated reference, and considering a confidence level of 95%, the statistic *t_exp_* can be evaluated, known to distribute as Student’s *t* with *q* + *n_cal_*–2 degrees of freedom:

(29) texp,j=HIT,c,j−HIT,calibratedu2HIT,c,j+u2HIT,calibrated~tq+ncal−2

(30) $$if\;t_{exp,j}\notin \left[t_{n_{1}+n_{cal}-2;0.025}^{};t_{n_{1}+n_{cal}-2;0.975}^{}\right]\to reject\;null\;hypothesis$$

where $t_{q+n_{cal}-2;0.975}^{}$ indicates the quantile of the $t_{q+n_{cal}-2}$ having a cumulative distribution of 0.975. The *t*-test on the group average can also be performed graphically by plotting error bars at a 95% confidence interval for the corrected value and the reference calibrated values. If the error bars overlap, then no systematic differences can be appreciated, with a risk of error of 5%.

Additionally, differences in terms of correction method precision can be investigated by a heteroskedasticity hypothesis test based on a χ^2^ distribution. First, representative corrective model variance $\sigma_{0}^{2}$ can be evaluated, from which the hypothesis test can be performed:

(31) $$u^{2}\left(H_{IT,c}\right)=\frac{\sum_{j=1}^{n}u^{2}\left(H_{IT,c,j}\right)}{n}\sim \chi_{q-1}^{2}\frac{\sigma_{0}^{2}}{q-1}$$

(32) $$\sigma_{0}^{2}=\frac{\sum_{m=1}^{M}u^{2}\left(H_{IT,c,m}\right)}{M}$$

(33) $$if\;u^{2}\left(H_{IT,c}\right)\notin \left[x_{teo;0.025}\frac{\sigma_{0}^{2}}{q-1};x_{teo;0.025}\frac{\sigma_{0}^{2}}{q-1}\right]\to reject\;null\;hypothesis$$

where $x_{teo;0.025}$ is the quantile of the $\chi_{q-1}^{2}$ distribution associated with a cumulated probability of 0.025.

Furthermore, the systematic significance of the accuracy can be investigated. This is relevant to understand whether, despite the pile-up correction, a bias is still left in the data with respect to the calibrated reference value. Assuming a null hypothesis such that there are no residual biases after the correction, i.e., the expected value of the accuracy is 0 GPa, let the score of the test be *x_exp_* which distributes as a χ^2^ distribution with a certain number of degrees of freedom *(dof*), and let $F_{\chi_{dof}^{2}}$ be its cumulative distribution function. The degrees of freedom can be evaluated by the Welch–Satterthwaite formula; see Equation (33) [85]. Then, at a 95% confidence level, the confidence interval can be evaluated, and the test performed:

(34) $$x_{exp}=nq\frac{{Acc}^{2}}{u^{2}\left(H_{IT,c}\right)+u^{2}\left(H_{IT,calibrated}\right)}\sim \chi_{dof}^{2}$$

(35) $$if\;x_{exp}>x_{teo},x_{teo}:F_{\chi_{dof}^{2}}\left(x_{teo}\right)=0.95\to reject\;null\;hypothesis$$

(36) $$dof=\left\lfloor \frac{{\left(u^{2}\left(H_{IT,c}\right)+u^{2}\left(H_{IT,calibrated}\right)\right)}^{2}}{\frac{u^{4}\left(H_{IT,c}\right)}{n(q-1)}+\frac{u^{4}\left(H_{IT,calibrated}\right)}{n_{cal}-1}}\right\rfloor$$

The test is performed considering a monolateral confidence interval because in the ideal condition, i.e., a perfect correction, the accuracy would be 0 GPa. This test allows a more synthetic and holistic overview than the pairwise *t*-test.

## 3. Results

**Figure 5:**
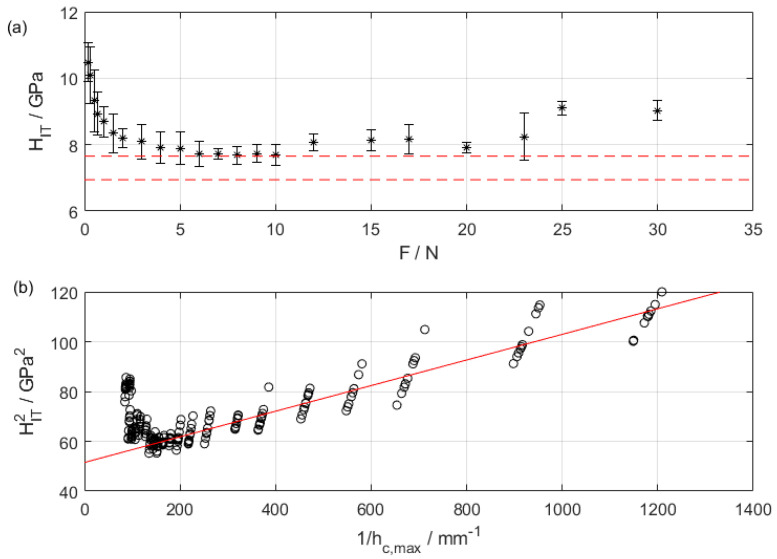
(**a**) Trend of *H_IT_* as a function of characterization force: notice the onset of ISE at forces smaller than 1 N, and pile-up leading to an overestimation of the hardness at forces larger than 10 N (error bars represent measurement uncertainty at a 95% confidence level). Red dashed lines are calibrated values. (**b**) Nix and Gao ISE modeling (red solid line) overlapping raw data: notice a strong deviation from linearity at small 1/h, i.e., at large penetration depths, indicating the occurrence of pile-up.

**Figure 6:**
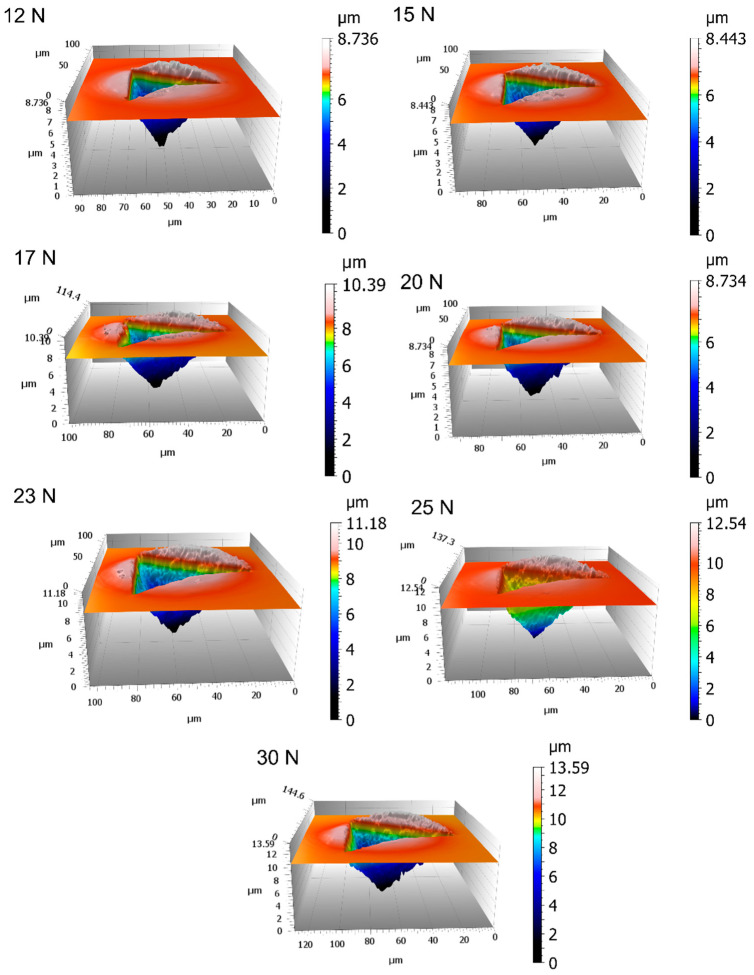
Measured surface topography of an indentation. Notice the relevant and not homogeneous pile-up.

Data collected as per the methodology described in Section 2.1 showed a raw trend of *H_IT_* indicating the presence of significant pile-up, leading to a systematic overestimation of the hardness by the calibrated contact area for forces larger than 10 N (see Figure 5a). Surface topographies of the indentations were measured by the CSI, highlighting severe pile-up at increasing load (Figure 6).

**Figure 7:**
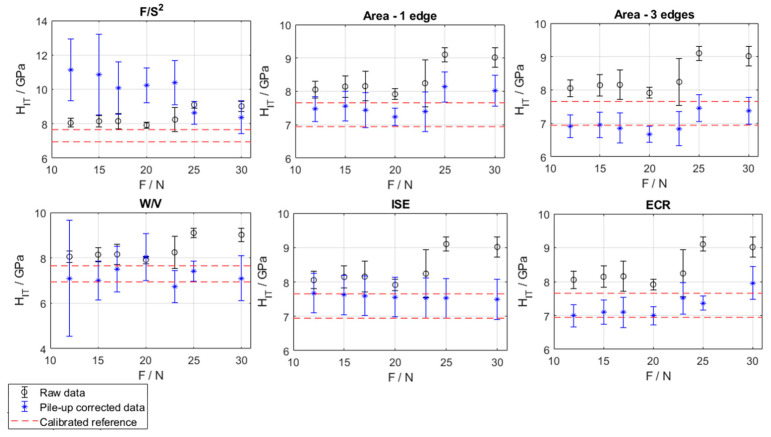
Results of the pile-up correction by the considered approaches: error bars at 95% confidence level. Black: raw data, blue: corrected data, red: calibrated values (upper and lower limit of confidence level at 95%).

Accordingly, the data were processed and corrected for pile-up using the methods described in Section 1.1. Figure 7 shows the results of the pile-up correction, considering the uncertainty propagation. The surface topography-based method exploiting geometrical characterization of the pile-up was applied considering only one, i.e., the maximum, pile-up edge, as well as all three edges.

Table 2 reports the accuracy and the standard uncertainty of the correction methods. Additionally, *p*-values of the hypothesis tests on the accuracy (i.e., to investigate the statistical relevance of the accuracy) and on the homogeneity of the methods’ dispersion (i.e., the heteroskedasticity test) are reported.

## 4. Discussion

Methods for pile-up correction selected from the literature were applied. The methodology innovatively introduced in Section 2.2 to propagate the measurement uncertainty of the correction and to subsequently assess the accuracy and the precision of the considered methods allows us to benchmark their performances.

As it can be seen in Figure 7, only the method based on the analysis of the indentation curve (F/S^2^) shows systematically poor performances in the correction at all considered loads, well summarized by the relatively high bias (2.84 GPa). Other methods do not present systematic differences, considering each force level, although some limit conditions, e.g., “Area—1 edge” at 25 N and 30 N, can be identified. Accordingly, and as expected, the correction of the surface-topography-based approach using all three edges (Area—3 edges) is more severe and leads to a better accuracy than the simplistic counterpart (Area—1 edge). Other methods (W/V, ISE and ECR) do not present systematic differences of the corrected values, thus showing good centering.

It is worth remarking that only the evaluation of the expanded uncertainty and the application of hypothesis tests allow determining within a metrological framework those conclusions that were not addressed in the literature.

In terms of accuracy (see Table 2), the best approach is ISE (0.28 GPa). For the more accurate methods, i.e., W/V, ISE and ECR, accuracy cannot be seen as a statistically systematic bias with a risk of error of 5%. Conversely, for methods based on surface topography geometrical characterization and the method based on the analysis of the indentation curve, a significant bias is shown with a confidence level of 95%

In terms of precision (see Table 2), among the most accurate methods, ECR is systematically more precise than others, having an expanded uncertainty of the corrected values of 0.376 GPa (evaluated at a confidence level of 95%). The surface topography methods considering all three indentation edges are also more precise than other pile-up correction methods. This result is consistent with the possibility of performing a traceable correction by means of an external reference. Conversely, the method based on the analysis of the indentation curve (F/S^2^) is also the least precise, showing a systematically larger variance.

Last, performance comparisons cannot neglect a further key feature of the presented methods, i.e., the practicality of the application. In fact, on the one hand, ISE is the most accurate, but it requires post hoc analysis to set up the regression. On the other hand, ECR can be considered. ECR has a slightly worse accuracy, which is still statistically not significant, and is the most precise approach. Once the system has been calibrated, ECR allows in-line correction of edge effects and predicts the hardness for the macro-range. In fact, ECR can continuously measure the area of a contact, which could be further used to evaluate the projected area. However, ECR can only be applied to conductive materials.

## 5. Conclusions

The Instrumented Indentation Test is a flexible and non-conventional hardness measurement technique allowing multi-scale and multi-scope mechanical characterization. The presence of edge-localized plasticity is extremely critical. This results in either sink-in or pile-up phenomena biasing the characterization typical for the most common industrially relevant materials. Therefore, several methods have been proposed in the literature to correct such systematic error.

This work reviewed the different approaches, proposing a classification while highlighting the main advantages and disadvantages. The main methods reported in the literature are based on work modeling, analysis of the indentation curve, topographical measurement of the edge-effect-affected surface (either exploiting areal measurement or volumetric measurements of the local plasticity), physical modeling of indentation size effect due to dislocation and data augmentation by in situ electrical contact resistance measurement.

Innovatively, this work proposed a metrological comparison of the methods’ performance, which is currently missing in the literature. In fact, although previous research typically benchmarked correction models, a holistic assessment considering measurement uncertainty and estimating metrological characteristics is often disregarded. The main results and conclusions that were obtained by the methodology introduced in this work to metrologically benchmark the considered pile-up correction methods are:

- different methods present significantly different metrological performances,
- Indentation Size Effect (ISE)-based modeling is more accurate (0.28 GPa with expanded uncertainty of 0.58 GPa),
- the data augmentation provided by the electrical contact resistance (ECR) shows the best precision (0.37 GPa) and second-best accuracy (0.33 GPa), and allows in-line correction, i.e., dispensing further post-processing,
- methods based on the analysis of the indentation curve (coupled with work-based modeling) and topographical measurements are suboptimal in terms of accuracy, leaving a systematic error after the correction.

Future work will focus on improving such approaches because they would allow a directly traceable correction. Additionally, an investigation of the correction performances at nanoscales, where optical resolution hinders application, by work-based approaches, ISE and ECR, which are the most promising, will be considered.

## Figures and Tables

**Table 1:** Summary review of edge-effect correction methods.

Edge-Effect Correction Method	Literature Sources	Notes
Work/Energy	[35,45,46,47,48,49,50,51,52,53,54,55,56,57]	From the fundamentals derived by Stilwell and Tabor [45], the seminal work of Sakai [50] was later modified and refined by Cheng and Cheng [57]. Effect of indenter geometry [53], materials and substrates for coating were more recently introduced [46,48].
Topographical measurement	Area evaluation [41,58,59,60]	Based on numerical evaluation from the Abbot–Firestone curve of a segmented portion of the indentation and edge-effect zones. The volume-based method also integrates work-based approaches originating from the work of Stilwell and Tabor [45].
	Volume evaluation [61]	
	Pile-up geometry measurement based on projected dimensions [62,63,64,65] or height [58]	The seminal work of Beegan et al. [62] was later modified and refined. Measurements typically rely on SPM [58,59,60,62,65,66,67] or optical instruments [41,61,63,64]. In some cases [62,63,66,67], profile measurements are considered, and the hypothesis of homogeneous pile-up is investigated [64,66]
Analysis of the Indentation Curve	[68,69,70,71]	Mostly relying on Cheng and Cheng’s work-based approach [57] and additionally exploiting the constant and area-independent ratio F/S^2^
Numerical Methods (FEM)	[72,73,74,75,76]	Largely exploited to address the inverse indentation problem to gather insight on material microstructure and localized plasticity.
Indentation Size Effect (ISE)	[33,77,78]	Nix and Gao proposed a model to predict and correct the ISE, but it also allows estimating a reference macro-hardness suitable to correct the pile-up.
Electrical Contact Resistance (ECR)	[79]	Exploiting data augmentation by in situ contact resistance measurement.

**Table 2:** Accuracy and average standard uncertainty of the correction methods. *p*-values of the hypothesis test on the systematic significance of the residual bias (accuracy) and on heteroskedasticity (systematic differences in precision). Significant *p*-values are summarized as * (<5%), ** (<0.1%), and *** (<0.01%).

Correction Method	Acc/GPa	$u\left(H_{IT,c,j}\right)$/GPa	*p*-Value Accuracy Test	*p*-Value Heteroskedasticity Test
**F/S^2^**	2.84	0.730	***	**
**Area—1 edge**	0.44	0.227	***	7%
***Area—3 edges***	*0.40*	*0.199*	**	* (3%)
**W/V**	0.39	0.433	>99.99%	57%
**ISE**	0.28	0.288	>99.99%	30%
**ECR**	0.33	0.188	18%	* (2%)

## Data Availability

Data available on request.
